# Simulation and Analysis of Road Traffic Noise among Urban Buildings Using Spatial Subdivision-Based Beam Tracing Method

**DOI:** 10.3390/ijerph16142491

**Published:** 2019-07-12

**Authors:** Haibo Wang, Ming Cai, Hongjun Cui

**Affiliations:** 1 School of Civil and Transportation Engineering, Hebei University of Technology, Tianjin 300401, China; 2 School of Intelligent Systems Engineering, Sun Yat-sen University, Guangzhou 510006, China

**Keywords:** road traffic noise, urban buildings, beam tracing method, building layouts, building heights

## Abstract

In order to realize the simulation and evaluation of road traffic noise among urban buildings, a spatial subdivision-based beam-tracing method is proposed in this study. First, the road traffic source is divided into sets of point sources and described with the help of vehicle emission model. Next, for each pair of source and receiver, spatial subdivision-based beam-tracing method is used in noise paths generation. At last, noise distribution can be got by noise calculation of all receivers considering the complex transmission among urban buildings. A measurement experiment with a point source is carried out to validate the accuracy of the method; the 0.8 m height and 2.5-m height average errors are about 0.9 dB and 1.2 dB, respectively. Moreover, traffic noise analysis under different building layouts and heights are presented by case applications and conclusions can be reached: (1) Different patterns result in different noise distributions and patterns designed as self-protective can lead to an obvious noise abatement for rear buildings. Noise differences between the front and rear buildings are about 7–12 dB with different patterns. (2) Noise value might not show a linear variation along with the height as shielding of different layers is various in reality.

## 1. Introduction

Urban and transportation development is having a huge effect on the human living environment. As one of the significant parts of traffic pollution, traffic noise has become an issue of concern in urban areas [1,2]. Constant exposure to noise will lead to health effects such as sleep disturbance [3,4,5], annoyance [6,7,8], cardiovascular effects [9], learning impairment [10,11], hypertension ischemic heart disease [12,13] and depressed mood [14]. Thus, it is important to avoid unwanted sound and particularly reduce reducing the noise exposure from the main sources: railway traffic [15], airports [16,17], industrial and ports [18,19] and road traffic [20]. Urban building areas which are usually with big road traffic, are the main places where people live and relax for most of the time. Therefore, noise simulation and evaluation in building areas are very important.

As the basic data of acoustical environment evaluation, the precise noise levels are usually derived from noise predictions in urban building areas because monitoring the noise in a large area is time-consuming and expensive [21]. However, modeling the sound field in a building area is difficult due to the complicated reflection and diffraction caused by buildings [22]. The current studies to calculate simulated noise focus primarily on the empirical method and the geometric acoustic method. The empirical methods such as ISO model [23] and its developing model [24,25], taking building density and the effects of front buildings into consideration, resulting in relatively large errors. Validated with reasonable accuracy and efficiency [26,27], the geometric acoustic method has been applied in traffic noise simulation, which consists of beam tracing method (BTM) [28,29], ray tracing method (RTM) [30,31] and image source method (ISM) [32]. With the assumption of sound waves are non-flexural, these methods are suitable in traffic noise simulation in urban building area as the wavelengths of traffic noise are much smaller than the building dimensions usually. Sound paths among urban buildings are complex, which consist of multiple reflections and diffractions. In such a situation, BTM can overcome the weakness of ISM’s limitation to diffraction calculation and the RTM’s sampling errors. Hence, BTM is widely used in noise simulation outdoors. For example, Wang et al. [26,33] and Luo et al. [34] presented beam tracing methods for traffic noise simulation. Considering the complex urban buildings environment, these studies avoid high computational complexity by pretreating the space.

Acoustical environment evaluation in an urban area has drawn huge attention of scholars. Based on monitoring or simulation data, noise distribution rules and pollution exposure with different dimensions are analyzed in recent decades. Such as: Cai et al. [35] suggested an approach considering high-resolution population and grid noise level to evaluate noise exposure in large urban area; Focused on building interiors, Funkhouse [36] presented a 3D model for architectural acoustics method; Hornikx et al. [37] predicted sound propagation in urban canyons and courtyards; Licitra et al. [38] presented comparative analysis of methods to estimate urban noise exposure of inhabitants, respectively. Additionally, the issue of noise distribution around urban buildings has received considerable attention. This argument is underpinned by the fact that some studies [39,40,41] indicate that building layouts, positions, heights have significant impacts on the acoustical environment.

The goal of this study is to simulation and analysis of road traffic noise among urban buildings. A method considering reflection and diffraction among irregular buildings using spatial subdivision-based beam-tracing method is presented. A building is chosen to verify the accuracy of the method. And the effects on noise distribution and rules caused by building layouts, heights are analyzed through the case applications.

## 2. Methods

### 2.1. Overview of the Method

**Figure 1:**
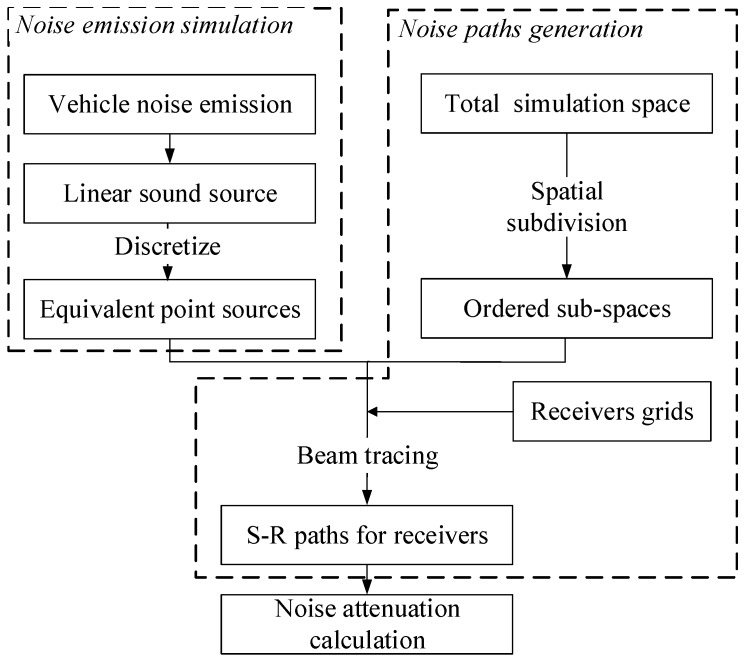
Overview of the noise calculation approach among buildings.

As shown in the Figure 1, the simulation of traffic noise among buildings can be divided into three distinct phases, which are traffic noise emission simulation, noise paths generation, and noise attenuation calculation. In the urban buildings area, the calculation region was set as a series of computational grids and the sound pressure level (SPL) of every note can be simulated. 

The procedure can be described as:

(1) For a road, it can be treated as a line source when considering the noise emission, and the line source can be equivalently divided into sets of point sources as the methods adopted in this study is available for the noise calculation between point sources and receivers.
(2) A spatial subdivision based on the Constrained Delaunay Triangulation (CDT) [42] was used for the acceleration of noise paths tracing and generation.
(3) The beam trees were built to store the necessary information via recursion, and all effective propagation paths from a noise source to a receiver point were found via the traversal of beam trees.
(4) For a pair of source and receiver, the noise attenuation among buildings was calculated considering the complex paths of direction, diffraction and reflection.
(5) The SPL of each receiver can be summed by the contribution of all the road segments.

### 2.2. Traffic Noise Emission Simulation

The traffic flow can be considered as an equal-value, linear sound source when the flow is sufficiently large according to relative study [43], and the vehicles on the road were treated as point sources which is omnidirectional. The vehicle noise emission is related to the types, speed, and acceleration of vehicles. Traffic noise also depends on the type of pavement, such as low-noise pavements are very effective in reducing noise [44]. An emission model of light, medium and heavy type vehicles based on the measurement data developed in China according to the Chinese standards [45] was adopted. Typical roads in China with a surface constructed of asphalt concrete are considered. The parameters of the surface can refer to the Chinese standard JTG F40-2004 (Technical Specification for Construction of Asphalt Pavements) [46]. The noise SPL $L_{0}(v)$ of a reference distance (7.5 m) of three types of vehicles with speed *v* can be described as

(1) $$L_{0}(v)=\{\begin{array}{cc} 27.96+24.92\mathrm{lg}(v), & \mathrm{Light}\;\mathrm{vehicle}\\ 16.44+36.73\mathrm{lg}(v), & \mathrm{Medium}\;\mathrm{vehicle}\\ 31.77+29.71\mathrm{lg}(v), & \mathrm{Heavy}\;\mathrm{vehicle} \end{array}$$

Considering the existence of the ground, the noise propagation of traffic noise in the surrounding urban area is hemispheric. And the noise power $W_{0}(v)$ of each type of vehicle at speed *v* is

(2) $$W_{0}(v)=2\pi \times 7.5^{2}\times 10^{0.1L_{0}(v)+12}$$

And the sound power $L_{l}(v)$ of the road with a *l* length and a *Q* traffic flow can be given by

(3) $$L_{l}(v)=W_{0}(v)\times \frac{Ql}{v}$$

**Figure 2:**
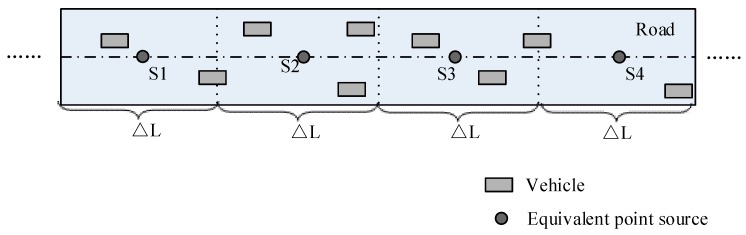
Discretization of a linear road source.

In this study, the road is discretized into a series of segments, as shown in Figure 2. The noise emission of each segment can be replaced by an energy equivalent point source and the location of the point source is set at the center of the segment. When coming to the noise calculation of point source and receiver, the segments can be regarded as independent ones. The sound power of the *i*-th segment with speed *v* can be described as

(4) $$L_{i}(v)=L_{l}(v)\times \frac{l_{i}}{l}=W_{0}(v)\times \frac{Ql_{i}}{v}$$

where, *l_i_* is the length of the *i*-th segment.

### 2.3. Noise Paths Generation Based on Spatial Subdivision

For the buildings area, the calculation area was subdivided into sets of subspaces with ordered numbers, with the goal of accelerating the paths generation process by searching the possible noise propagation in a local area. The triangular prism was chosen as the basic spatial subdivision cell. The spatial subdivision is based on CDT, and the triangular prisms are determined by the layout of buildings and noise sources.

According to previous studies [47,48], the frequency of traffic noise is concentrated in the range of 500 Hz–2.5 kHz. The wavelength of traffic noise primarily ranges from 13.6–68 cm, which is smaller than the outdoor surface dimensions. Hence, the geometric acoustic method was adopted in this paper assuming that the sound waves are non-flexural, which means that noise propagates along straight paths among outdoor building areas.

In reality, most buildings with an irregular basis are successive. The beam tracing method (BTM) is the most suitable option for noise paths generation in this situation. With a certain spatial subdivision, a tree structure was adopted to store the ordered beams for every source. For each receiver, the beam tree structures of every source were traced in reverse, and records of the influenced subspaces and edges or façades that are passed by the beam were acquired. The node of the tree structure includes the subspace code, the reflection time, the diffraction time, the source location, etc. Paths can be generated with the help of tree structure. For each receiver and source pair, the paths between them can be continually acquired by the tree structure as follows: find the subspace containing the receiver, enumerate all the nodes in the structure and seek the target nodes meeting the requirement until the end. During the generation process, records of the influenced subspaces and edges or façades that the beam passes are acquired. When the target nodes are found, an effective 3D path consisting of reflections and diffractions from the source to the receiver is obtained.

Obviously, for a pair of source and receiver, the noise path is not unique. The sound paths may consist of directed ones, reflect ones, diffract ones or complex ones. During the path generation process, if one of the three following conditions is satisfied, the tracing was complete:

(1) The length of the beam reaches the desired maximum value.
(2) The reflection or diffraction time exceeds the desired maximum value.
(3) The beam reaches the boundary of the calculating space.

### 2.4. Noise Calculation of Receivers

The noise propagates to the complex urban environment by different sequences of reflection, transmission, and diffraction. At any time t, the sound intensity at point P caused by the *i*-th vehicle is the sum of all propagation paths, as follow:

(5) $$I_{P(i,t)}=I_{D(i,t)}+I_{R(i,t)}+I_{Diff(i,t)}+I_{Com(i,t)}$$

where *I*_*D*(*i*,*t*)_, *I*_*R*(*i*,*t*)_, *I*_*Diff*(*i*,*t*)_ and *I*_*Com*(*i*,*t*)_ are, respectively, the contributions of the direct transmission beam, the reflected beam, the diffracted beam, and the composite beam. The specific calculations for the four types of beams can be found in [34].

Hence, the SPL of a receiver can be given as

(6) $$L_{p}=10\mathrm{lg}(\sum_{i=0}^{n}I_{Pi}\cdot \rho_{0}C/p_{0}^{}{}^{2})$$

where $\rho_{0}$ is the density of air (in kg/m^3^), C is the noise speed (in m/s), and $\rho_{0}C$ is the air’s acoustical impedance, which can be set to 415 N·S/m^2^ at a temperature of 20 °C.

## 3. Validation of the Method

### 3.1. Experiment Description

**Figure 3:**
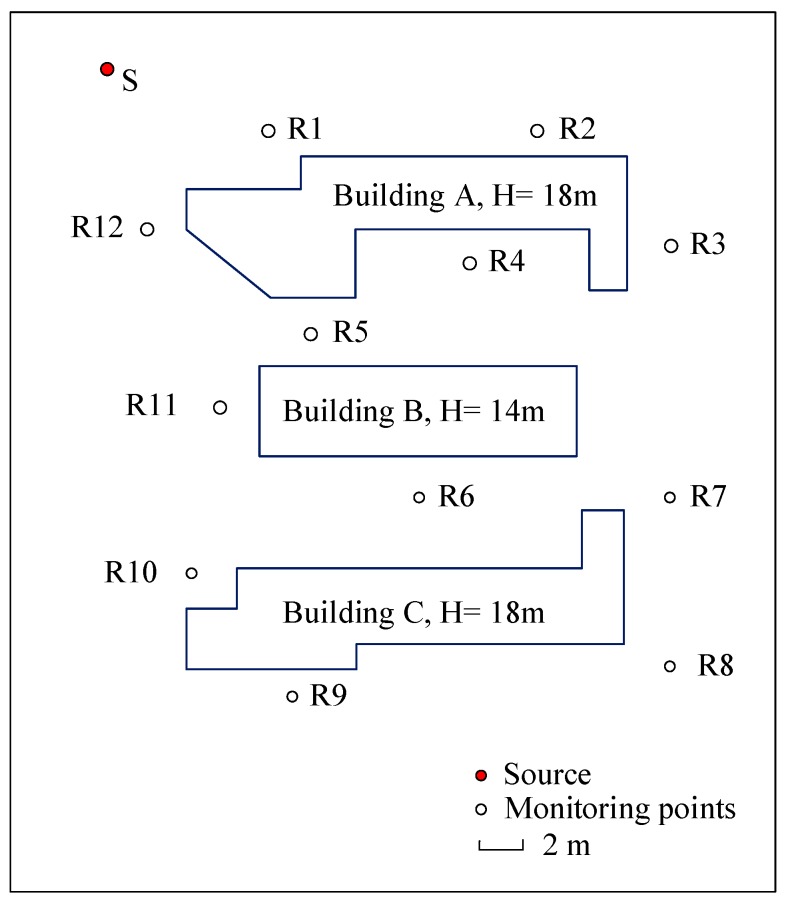
Sketch of the experiment.

An experiment of noise attenuation among buildings from a point source was carried out to validate the accuracy of the method. The area at Hebei University of Technology in Tianjin, in which there are three buildings, was chosen to implement the validation experiment. The temperature was 20 °C, the humidity was 17%, and the average wind speed was 0.2 m/s. The measurements complied strictly with the relevant Chinese standards and specifications. The buildings (A, B, C) layout, the positions of monitoring points (numbered with 1–12) and source (S) are shown in the Figure 3. The ground is made of asphalt and the estimated reflection coefficient of the buildings and asphalt ground is about 0.85. The background noise is measured with 38.7 dB. Due to the complexity of the frequency, the dominant frequency of 630 Hz, which represents the primary frequency of traffic noise, was selected for experiment and noise simulation calculation. The height of the point source in the experiment is 0.8 m, which emitted by an omnidirectional fixed frequency generator of 630 Hz in 1/3 octave band. The reference noise value (7.5 m away from the source) is 83.2 dB. Noise data at 12 points with two heights (0.8 m, 2.5 m) were collected, and AWA6228 (Hangzhou Aihua Instruments Co., Ltd., Hangzhou, China) sound level meters were used in the process.

When coming to the noise simulation calculation, the reflection diffraction thresholds were both set as 10; the length threshold of the beam was set as 500 m. This setting ensures enough reflections and diffractions combination for these building configurations.

### 3.2. Analysis of Results

The sound measurement and calculation results of 12 receivers with two heights are shown in Table 1.

**Figure 4:**
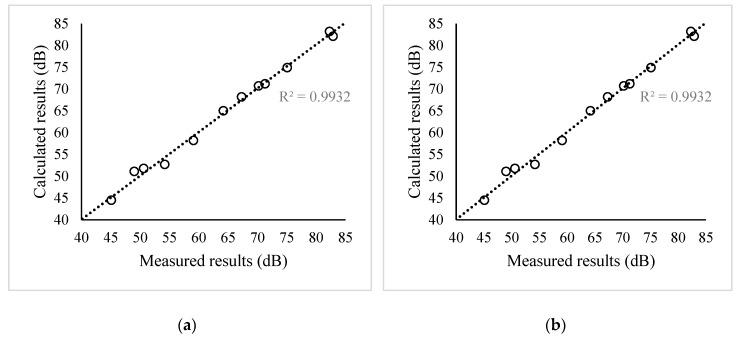
The correlation analysis of the measured and calculated data. (**a**) H = 0.8 m; (**b**) H = 2.5 m.

In the validation experiment, the average absolute errors of the noise values between measured and calculated for 0.8 m and 2.5 m are about, respectively, 0.9 dB and 1.2 dB. The largest error was found at point 4 at 0.8 m height and point 8 at height of 2.5 m, and the measured data deviated from the calculated data by 2.1 dB and −2.7 dB, respectively. Figure 4 presents the correlation analysis of measured and calculated data of 12 points with two heights.

Table 1 and Figure 4 show the high correlation between measured values and calculated values. The Pearson correlation coefficients of these two heights are 0.9932 and 0.9876, respectively, which are nearly 1, indicating the good performance of this model. The tendency of the calculated values along with the height is generally in agreement with the measured values. It shows that this model performs well in reflecting the distribution pattern of the height, although the accuracy decreases slightly as the height increased.

**Figure 5:**
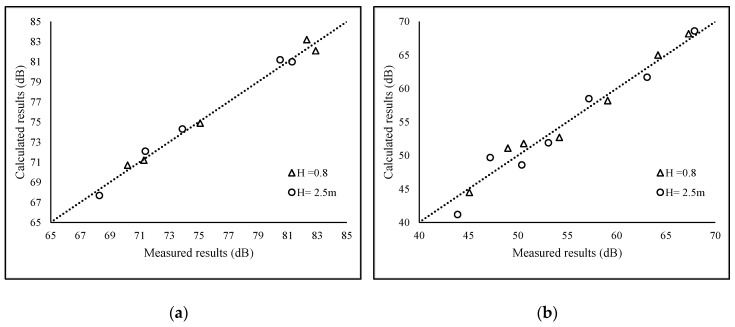
The correlation analysis of the measured and calculated data. (**a**) Direct transmission and reflection calculation; (**b**) diffraction or composition beam calculation.

Figure 5 shows the correlation analysis of the measured and calculated data from the aspect of the main propagation of sound. Although the direct transmission and reflection calculation (with a 0.9964 Pearson correlation coefficient) has better accuracy compared with the diffraction or composition beam calculation (with a 0.9786 Pearson correlation coefficient), the composition beam calculation still keeps a satisfying performance.

There are nine point-height data with calculated errors above 1 dB (points 4, 7 and 9 at 0.8 m height; points 3, 4, 6, 7, 8 and 9 at 2.5 m height). Their common characteristic is that the sound paths are complex and include multiple reflections and diffractions. The reason is that with the noise propagation, the process becomes more complicated and the errors accumulate. 

Overall, these results demonstrated that this model generally works effectively for noise propagation to buildings in the 3D external space because of its high accuracy.

## 4. Application and Discussion

The existence of buildings has a big affection on the urban noise levels. In this study, the influences of building layouts and unequal height buildings on traffic noise were simulated and discussed. In this section, the reflection coefficient of the outdoor wall was set as 0.85, and the times of reflection and diffraction were set as 10. The length threshold of the beam was set as 500 m.

### 4.1. Effect of Building Layouts in Urban Environment

Because buildings occlude and reflect traffic noise, the layout of the building group has an obvious influence on the sound field of the area. Simulations and comparisons of traffic noise with different buildings layouts are presented in this section, and the advantages and disadvantages of different layouts for noise abatement are analyzed.

#### 4.1.1. Settings with Different Layouts

**Figure 6:**
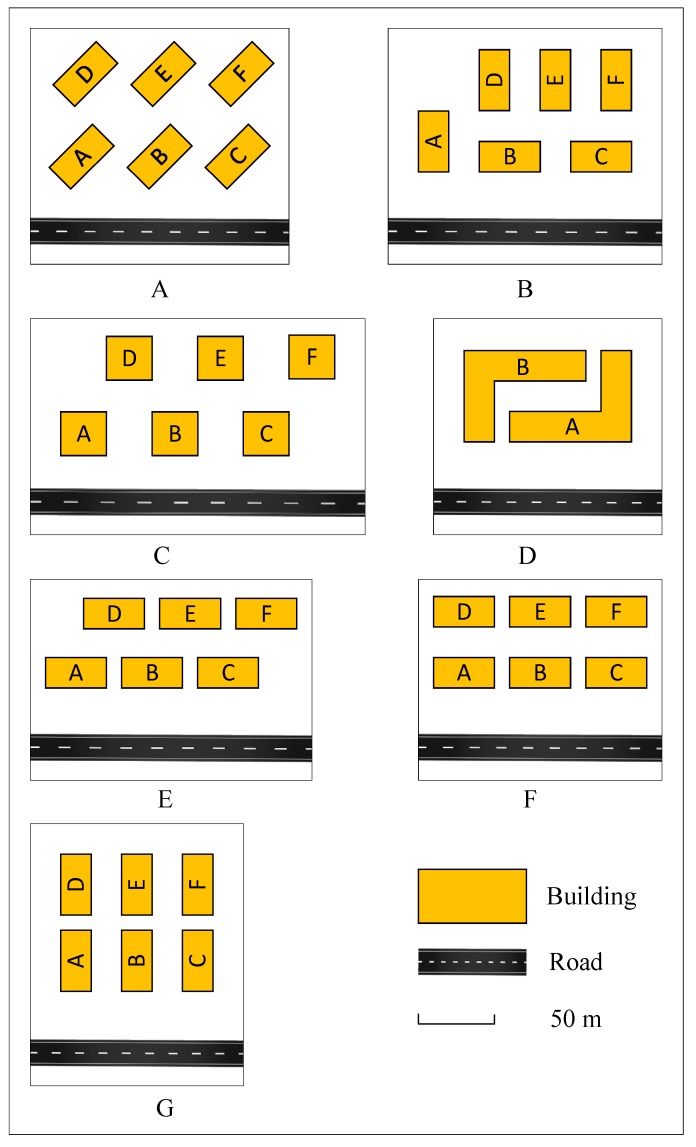
Seven layout patterns. (**A**) Pattern A; (**B**) Pattern B; (**C**) Pattern C; (**D**) Pattern D; (**E**) Pattern E; (**F**) Pattern F; (**G**) Pattern G.

As shown in Figure 6, seven layout patterns were considered. The underlying parameters are the same or similar for each pattern. The total area of the buildings is 4800 m^2^, and the height of each building is 42 m. The distance between the fronts of the buildings and the road is 30 m. The frontal distance between buildings is 20 m, and the lateral distance between buildings is 10 m.

In this experiment, a two-lane road was considered on which the average speed is 60 km/h and the traffic flow is 1200 vehicles per hour. The calculated spacing of the receivers is 3 m, and the background noise is 40 dB. 

#### 4.1.2. Analysis of Calculation Results

**Figure 7:**
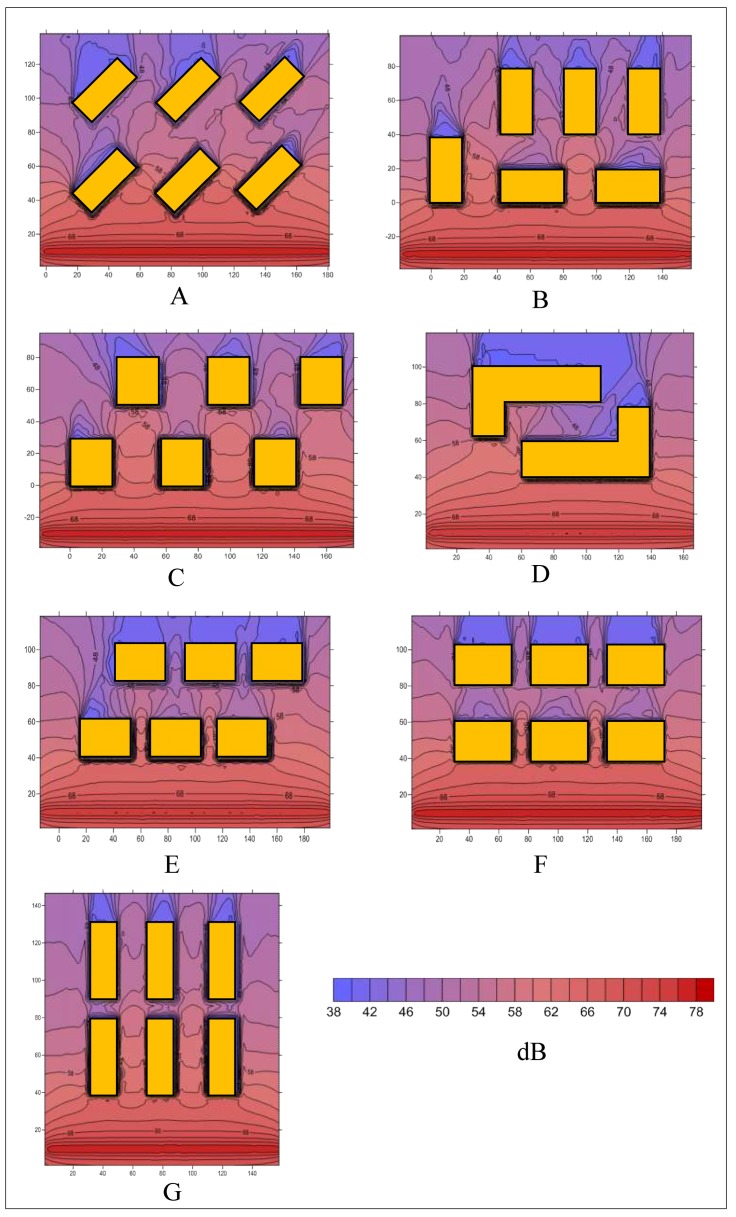
Noise distributions of seven patterns at an 8-m-high plane. (**A**) Pattern A; (**B**) Pattern B; (**C**) Pattern C; (**D**) Pattern D; (**E**) Pattern E; (**F**) Pattern F; (**G**) Pattern G.

The noise values of different patterns are calculated by the proposed method. Figure 7 shows the noise distributions at an 8-m-high plane.

**Figure 8:**
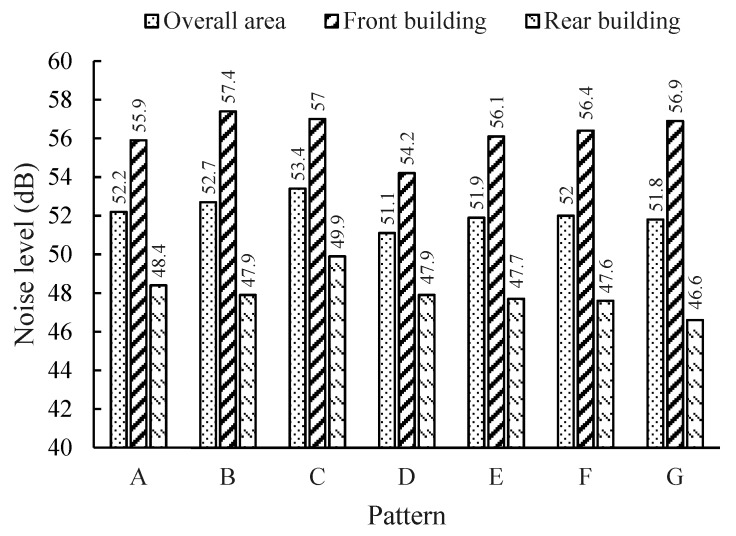
Noise levels for different patterns (in dB).

Traffic noise mainly propagates to indoor areas of a building through the façade; hence, the average noise levels of buildings’ façades, in the unit area, were chosen to assess the noise pollution. The statistical results of the overall area, the front buildings and the rear buildings of the seven patterns were calculated and are shown in Figure 8.

It can be indicated in Figure 7 and Figure 8:

(a) The noise level of Pattern C (53.4 dB) is much higher than that of the other patterns in terms of an overall area, because parts of the second-row buildings are source-direct. Pattern D outperforms the other patterns by at least 0.7 dB. The almost-enclosed region leads to a relatively quiet environment.
(b) The noise pollution in the front buildings of all the patterns is more serious. However, although they share the same distances between the front buildings and the road, the noise levels are different for different patterns. According to the statistics, the noise level of Pattern B is the highest (57.4 dB), followed by those of Pattern C, Pattern G, Pattern F, Pattern E, and Pattern A. The noise level of Pattern D is the lowest, at 54.2 dB.
(c) For the rear buildings, the most serious noise pollution appeared in Pattern C (49.9 dB), where the noise level was at least 1.5 dB higher than those of the other patterns. The difference between the noise levels of the other patterns is not large—approximately 48 dB. Pattern G (46.6 dB) performed the best when only rear buildings are considered.
(d) For the seven patterns, the maximum (10.3 dB) and minimum (7.1 dB) noise differences between the front and rear buildings appeared in Patterns G and C, respectively.

Compared to other types of pollution, an important feature of noise pollution is that the spatial variance is significant, even at the very small scales of urban texture and transportation infrastructure [49]. Therefore, it is important to plan building layouts strategically. Building layout patterns can be designed to be self-protective from traffic noise. Consider Pattern D as an example: The front buildings, which suffer from serious noise pollution, can be used for commerce. The front buildings act as noise barriers for the rear buildings, which can be primarily residential.

### 4.2. Noise Distribution Effect of Heights of Buildings

Actually, the heights of buildings are various in urban area, which have a big effect on noise distribution. What’s more, due to the relative positions between buildings and roads, front or sheltered buildings with the same height may also have different influence on noise attenuation. The area of Tianjin Quanyun District in Tianjin was chosen to study the noise distribution effect of buildings heights. As a typical form of cell layout, Tianjin Quanyun District can be regarded as Pattern F or Pattern G, in which the noise levels of inner buildings are comparatively low. For accurate research (with determined information of roads, traffic and buildings) and practical guidance, a real case was considered to analyze the noise distribution effect of heights of buildings. With the initial assumption that both the two applications are with universality noise emission model and regular buildings, Tianjin Quanyun District can also be treated as an ideal scene.

#### 4.2.1. Study Site

**Figure 9:**
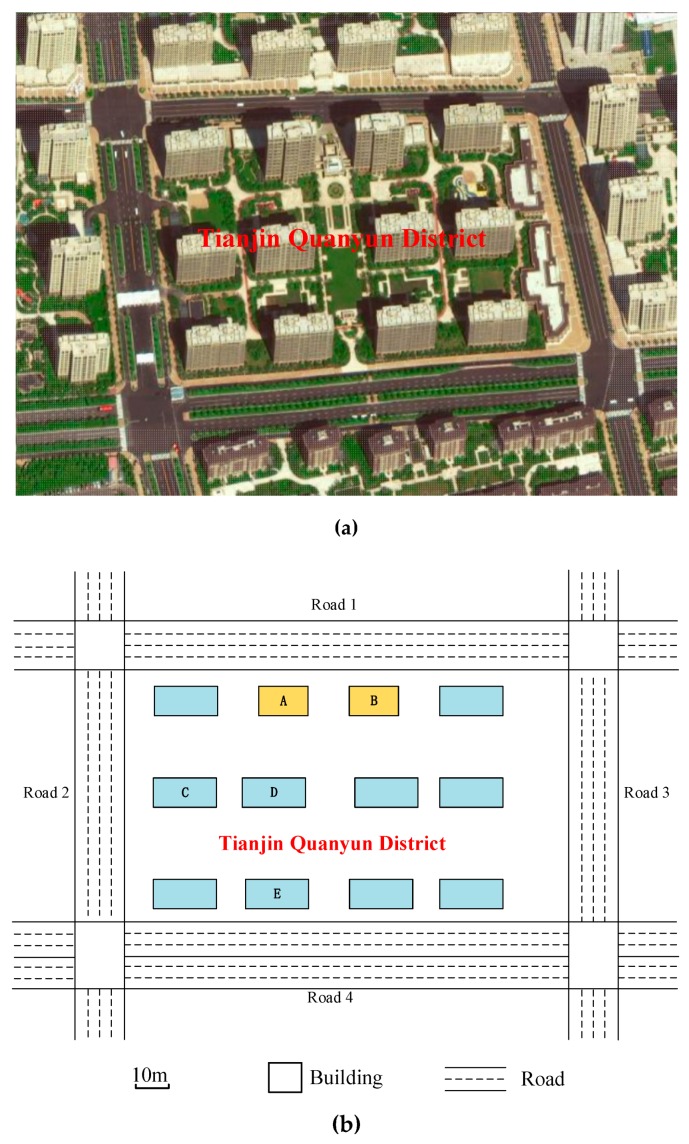
Configuration of study case. (**a**) Map of Tianjin Quanyun District; (**b**) road network and building information.

The road lanes, road network, building layout and dimensions are shown in the Figure 9a. And the roads were considered on which the average speed is 60 km/h and the traffic flow is 800 vehicles per hour every lane. The background noise is 40 dB. There are two types of buildings. The horizontal surface area of Type I (building A and building B) is 35 × 10 m^2^, with the height of 42 m. And the horizontal surface area of Type II (other buildings) is 42 × 10 m^2^, with the height of 27 m. Two types of buildings are marked yellow and blue in Figure 9b, respectively.

**Figure 10:**
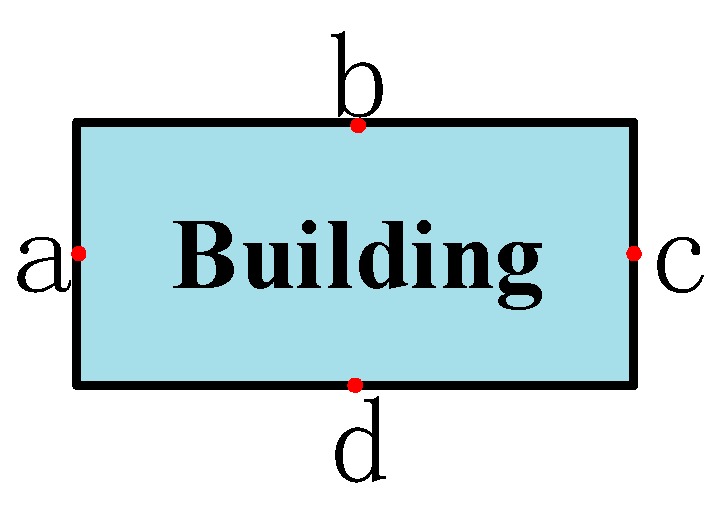
Depiction of chosen lines for noise calculation.

To discover the sound attenuation effects of different height, buildings A, C, D and E were chosen. As indicated by points in Figure 10, the noise level of four vertical lines a, b, c and d, which is at the middle of different façade centers, are calculated for noise analyses.

#### 4.2.2. Analysis of Calculation Results

**Figure 11:**
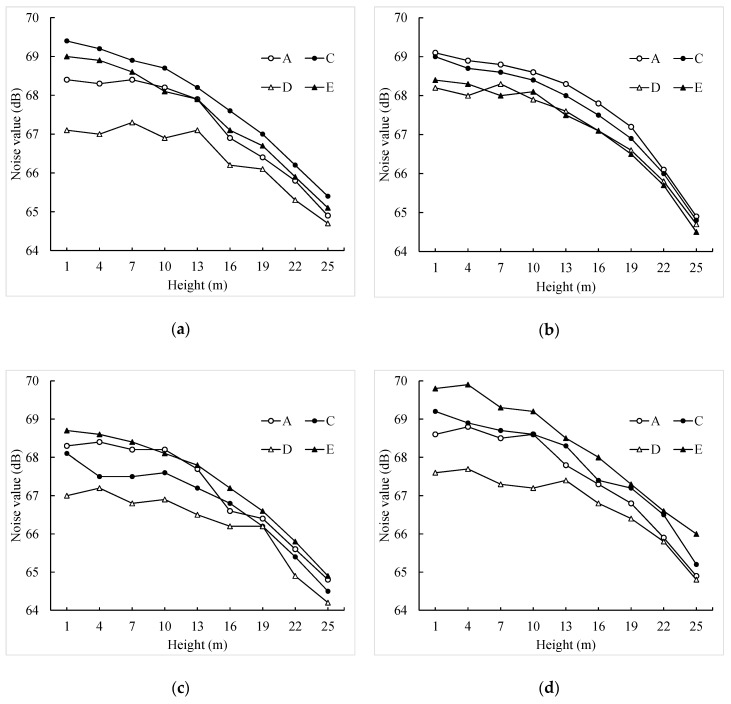
Noise value of different planes. (**a**) Façade a; (**b**) façade b; (**c**) façade c; (**d**) façade d.

The noise values of some equidistant ordinates on chosen lines a, b, c and d are, respectively, drawn in the line charts of Figure 11.

As shown in Figure 11, the traffic noise level, almost at all façades, render the propagation of sound attenuation rules well. Although the noise level decrease with the height increase, the traffic noise value might not show a linear variation along with the building height. It might be fluctuant, which is consistent with the perceptions of the residents. Because buildings are in different heights in the area, which means that the shielding of different layers is various. When below the height of 15 m, the noise level of the upper layers could be impacted by the additional sources of road traffic noise, which might lead to the sum of the noise impact on the upper layers being higher than that on the lower ones. The outdoor traffic noise level clearly shows that the noise level declines because of the shielding effect of the building and the long distance between the receivers and the sources. For example, the noise values of façade b and c of building D are almost the least compared to the other façades or buildings at the same height.

Avoiding the purchase of a flat on a lower level is recommended if only taking the height into consideration. But in reality, the situation is more complicated—consideration of the shielding of street-face buildings on a different floor is more reasonable.

## 5. Conclusions

To the simulation and evaluation of road traffic noise among urban area, a universal 3D calculation model of road traffic noise propagation among urban buildings using a beam tracing method which is based on spatial subdivision is presented in this paper. The method realizes noise calculation by the processes of noise emission, paths generation and noise calculation with the help of tree structures. Meanwhile, spatial subdivision of the calculation area is adopted with the goal of accelerating the path generation process by searching the possible noise propagation in a local area. In this study, typical roads in China with a surface constructed of asphalt concrete are considered for universality. Research on types of pavement, such as low-noise pavements, is recommended for future work.

The accuracy of the method is validated by an experiment with average errors of about 0.9 dB and 1.2 dB, respectively, at the 0.8-m height and 2.5-m height. Although the errors accumulate as the noise propagation process become more complicated, the results demonstrated that this model generally works effectively for noise propagation to buildings in the 3D external space because of its high accuracy.

The effects of outdoor building layout and building height on traffic noise were simulated using the proposed method and the results were discussed. The results show that building layout patterns and building heights play large roles in distributing traffic noise. In the study cases, although the noise values in the front buildings are much higher than those in the rear buildings, with about 7–12 dB D-value of different patterns, different patterns result in different noise distributions rules. Patterns designed as self-protective can lead to a more obvious noise abatement for rear buildings, such as Pattern G in this study. Moreover, noise value might not show a linear variation along with the height as shielding of different layers is various in reality. When below the height of 15 m, the noise level of the upper layers is impacted by the additional sources of road traffic noise, which leads to an obvious noise superposition effect compared with the lower ones.

This paper includes a road traffic noise evaluation into the design of a residential area. Moreover, for a more livable environment, residents can choose the quieter floor and flat orientation and arrange the flat layout referring to the simulated results. These applications have expanded the practical utility of this model.

## Figures and Tables

**Table 1:** Comparison of measured and calculated results (in dB).

Point	Main Propagation	H = 0.8 m			H = 2.5 m		
		Measured	Calculated	Deviation	Measured	Calculated	Deviation
1	Direct Transmission & Reflection	82.9	82.1	−0.8	81.3	81.0	−0.3
2	Direct Transmission & Reflection	71.3	71.2	−0.1	71.4	72.1	0.7
3	Diffraction	64.2	65	0.8	63.1	61.7	−1.4
4	Composition Transmission	49.0	51.1	2.1	47.2	49.7	2.5
5	Composition Transmission	67.3	68.2	0.9	67.9	68.6	0.7
6	Composition Transmission	59.1	58.2	−0.9	57.2	58.5	1.3
7	Composition Transmission	50.6	51.8	1.2	50.4	48.6	−1.8
8	Composition Transmission	45.1	44.5	−0.6	43.9	41.2	−2.7
9	Diffraction	54.2	52.7	−1.5	53.1	51.9	−1.2
10	Direct Transmission & Reflection	70.2	70.7	0.5	68.3	67.7	−0.6
11	Direct Transmission & Reflection	75.1	74.9	−0.2	73.9	74.3	0.4
12	Direct Transmission & Reflection	82.3	83.2	0.9	80.5	81.2	0.7
